# Effectiveness of a Standardized Combination of Intracameral Mydriatics and Anaesthetic on Mydriasis during Cataract Surgery with Coexisting Diseases

**DOI:** 10.3390/life14010014

**Published:** 2023-12-21

**Authors:** Joanna Katarzyna Dereń-Szumełda, Mariola Dorecka, Łukasz Zandecki, Ewa Mrukwa-Kominek

**Affiliations:** 1 Military Institute of Aviation Medicine, Krasińskiego 54/56, 01-755 Warszawa, Poland; 2 Department of Ophthalmology, Faculty of Medical Sciences in Katowice, Medical University of Silesia, 40-514 Katowice, Poland; mdorecka@sum.edu.pl; 3 Collegium Medicum, Jan Kochanowski University of Kielce, Żeromskiego 5, 25-369 Kielce, Poland; lukasz.zandecki@gmail.com; 4 Faculty of Medical Sciences in Katowice, Medical University of Silesia, Poniatowskiego 15, 40-055 Katowice, Poland; ewa.mrukwa@sum.edu.pl; 5 Department of Ophthalmology, Kornel Gibiński University Clinical Center, Ceglana 35, 40-514 Katowice, Poland

**Keywords:** cataract, mydriasis, diabetes, pseudoexfoliation syndrome, eyedrops

## Abstract

Purpose: To examine the effectiveness of a standardized combination of intracameral mydriatics and anesthetic (SCIMA) on mydriasis in patients with coexisting diseases such as diabetes mellitus (DM) and pseudoexfoliation syndrome (PXF) during phacoemulsification. Methods: Patients with cataract were included in the study if they achieved pupil dilation diameter ≥ 6.0 mm after the administration of mydriatic eyedrops (ME) during the first visit (V1). During the second visit (V2), pupil size measurements were obtained for phacoemulsification surgery with SCIMA. Effective mydriasis was defined as a pupil diameter ≥ 6.0 mm just prior to capsulorhexis without the use of additional pupil dilating agents. The measurements after ME administration during V1 and after SCIMA use during V2 were compared. Results: 103 patients (103 eyes) were divided into 3 groups: cataract and DM (*n* = 35), cataract and PXF (*n* = 32), and cataract without DM or PXF (*n* = 36). SCIMA administration allowed the achievement of effective mydriasis (≥6.0 mm) in all groups (*n* = 103; 100%). Mydriasis was significantly larger (*p* ≤ 0.001) after ME (7.3 mm) than after SCIMA (6.8 mm) administration. Conclusions: Patients with cataract and such comorbidities as DM or PXF are likely to achieve effective pharmacological mydriasis during cataract phacoemulsification after SCIMA application. Mydriasis after ME is slower and larger, while SCIMA is faster.

## 1. Introduction

The rising incidence of age-related diseases has been driven by an increase in life expectancy, and therefore, significantly more cataract cases are expected in the general population in the coming years [1,2]. Lens opacity, commonly referred to as cataract, is a curable disease, and yet, on a global scale, it remains one of the leading causes of blindness, according to the World Health Organization (WHO). It is estimated that approximately 51% of all blindness cases worldwide, totaling around 21 million people, are due to cataract. Notably, approximately 90% of individuals afflicted by blindness caused by cataract are from developing countries [3]. Cataract, is a curable condition that requires surgical removal of the opaque lens, which is currently its only treatment. According to estimates, developed countries see over 10,000 cataract surgeries per million inhabitants each year [4]. According to National Health Fund (NFZ) statistics, 72.40% of the patients who undergo surgery in Poland are in the 61–80 age group [5]. For instance, in England, between 2017 and 2018, over 398,000 cataract surgeries were performed using phacoemulsification, making cataract surgery the most frequently conducted planned surgical procedure [6]. Currently, microincision phacoemulsification is the most preferred method for cataract extraction. The duration of the procedure typically ranges from 10 to 20 min and can vary based on factors such as the cataract type, the surgeon’s experience, and the presence of any concurrent eye conditions, particularly ocular pathologies [7]. Ensuring the optimal pupil diameter throughout the procedure and effectively managing inflammation of the eyeball play a vital role in attaining the desired surgical outcomes. It is important that the pupil maintains a size of ≥6.0 mm during the procedure, especially at the time of the key stages of the surgery: capsulorhexis, phacoemulsification, and lens implantation. Maintaining the specific distance from the pupillary margin facilitates the precise creation of a 5.0 mm wide capsulorhexis under visual control, ensuring it is centrally located, the right size, and perfectly round. In fact, one approach suggests that with a 6.0 mm pupil diameter, half of the intra-surgical complications can be reduced [8]. Effective mydriasis and anesthesia during cataract surgery are the basic conditions for the surgery to be safe, performed correctly, and with the highest possible comfort for the patient [9,10].

Both ophthalmic diseases such as glaucoma, as well as systemic comorbidities, like diabetes mellitus and pseudoexfoliation syndrome (PXF), and the use of non-selective α1 adrenergic receptor antagonists (α1-blockers) for treating prostatic hyperplasia or arterial hypertension, may pose challenges in dilating the pupil [11].

The most common civilization disease in the world is DM. As in the case of cataract, it is estimated that there will be a significant increase in its incidence in the coming years [12]. DM comprises a group of metabolic disorders characterized by elevated blood glucose levels due to issues with insulin secretion and/or insulin function. Prolonged high blood glucose levels can lead to organ damage, including the eyes. Diabetic eye diseases are considered to be severe as they can result in reduced vision and, in advanced stages, even blindness. The most important entities include diabetic retinopathy, secondary glaucoma, and cataract [13]. It has been assessed that up to 20% of all cataract surgeries are performed in diabetic patients in whom this disease may lead to a smaller pupil dilation [14]. Poor mydriasis in diabetes is usually attributed to disruptions in iris innervation and the loss of mechanical properties in its stroma [15].

The influence of PXF on the iris tissue is also reflected in the clinical aspect of cataract surgery [16,17]. PXF is an age-related systemic condition where extracellular fibrillar material accumulates in various tissues throughout the entire body [18]. Furthermore, this is clearly evident in the anterior chamber of the eye. The accumulation of elastic fibers in the stroma or muscle tissue, along with vascular abnormalities, results in hypoxia and tissue degeneration. Consequently, the iris in PXF syndrome manifests characteristic rigidity, accompanied by a diminished ability to dilate [19]. 

Iris changes in both diseases are important risk factors in cataract surgery as they have a negative impact on achieving the optimal pupil diameter [16,20].

The mydriasis required for ophthalmic surgery is usually obtained by topical drugs and/or injections into the anterior chamber. These include α1-adrenergic agonists with sympathomimetic effects, known as sympathomimetics, and cholinergic muscarinic antagonists with parasympatholytic effects, referred to as cholinolytics [21]. They are used either individually or in combination. Conventionally, before cataract surgery to induce pharmacological mydriasis, standard practice involves the application of topical mydriatic eyedrops (ME) three times at 5 min intervals. These drops usually contain 1% tropicamide and/or 10% phenylephrine. The use of pupil-dilating drugs with different points of action makes it easier and faster to achieve the desired mydriatic effect. This is known as a superadductive mechanism—the dilator muscle of the iris is stimulated simultaneously while its antagonist, the constrictor muscle, is blocked [21].

However, the constant pressure of time in the operating room conditions and the need to minimize the time of medical procedures made it necessary to start searching for new methods and therapeutic solutions to improve the process of perioperative activities, including preparation for surgery, the procedure itself, and postoperative care.

An innovative solution for administering mydriatics to the ocular surface, reducing the time required for drug administration by medical personnel, decreasing the drug dosage, and minimizing adverse effects (as observed in the case of ME), is represented by Mydriasert [22]. It is a non-soluble ophthalmic insert designed for pre-surgical mydriasis, placed 60 min before surgery in the lower conjunctival fornix of the operated eye. It gradually releases the active ingredients, tropicamide (0.25 mg) and phenylephrine (5.38 mg) [23]. In the conducted studies, it was observed that the mydriatic effect of Mydriasert is comparable to ME after 60 min and surpasses them after 90 min [24].

In 2015, a standardized combination of mydriatics (0.02% tropicamide, 0.31% phenylephrine) and an anesthetic (1% lidocaine) [25] (SCIMA)—Mydrane—was introduced in the form of intracameral injections to obtain mydriasis during cataract surgery. One 0.2 mL dose contains 0.04 mg of tropicamide, 0.62 mg of phenylephrine hydrochloride, and 2 mg of lidocaine hydrochloride [11,26]. The mydriatic effect of those combined substances in one solution is greater than that of any of these agents alone [27].

During phase II [28] and phase III [29] studies on Mydrane, it was found that the combined doses of active substances provided safety and efficacy. It was also confirmed that the dose of Mydrane exhibited a similar effectiveness to standard mydriatics [29] while containing lower concentrations of active substances, which may reduce the risk of adverse events and increase the safety of the procedure [15,30].

However, there are no exact data on the use of Mydrane in certain groups of patients with systemic comorbidities and the risk of pupil narrowing during the surgery, including patients with DM (treated with insulin or oral medications) and PXF [25,27,28].

The study aimed to assess whether the use of an appropriate combination of mydriatics with an anesthetic in Mydrane would provide effective pharmacological mydriasis in patients with cataract coexisting with such general diseases as DM and PXF, as it does in cataract patients without those diseases.

## 2. Methods

This paper presents a real-life study without randomization. The observational, non-interventional study followed the tenets of the Declaration of Helsinki. Written informed consent was obtained from each patient after explaining the nature and potential consequences of the study. The study was approved by the Ethics Committee and was conducted on 6–24 March 2017, 15–29 March 2018, and 28 June–16 July 2019 at the University Clinical Centre, Medical University of Silesia, Katowice, Poland. 

### 2.1. Patients

Patients were included in the study if they achieved a pupil dilation diameter ≥ 6.0 mm after the administration of ME in the initial examination in accordance with the Summary of Product Characteristics. The inclusion and exclusion criteria only qualified patients with an optimal response to ME (1% tropicamide and 10% phenylephrine) and disqualified patients whose results in the initial examination might have influenced the analysis of the results (combined incidence of DM and PXF, use of α1-blockers, damaged iris structure, use of additional pupil dilation agents or stabilizing devices).

It is worth noting that the exclusion criteria used in this study, with the exception of the limitations included in the product characteristics summary, do not constitute a significant limitation in everyday medical practice. Furthermore, guided by ethical considerations and prioritizing patient well-being and safety, at each stage of the operation, the surgeon had the opportunity to use additional measures to dilate and stabilize the pupil, such as adrenaline or iris retractors, when local conditions and the safety of the procedure necessitated it. Any additional procedures or substances introduced during cataract surgery beyond the standard protocol were meticulously documented. When additional methods were used to enhance pupil dilation, only pupil diameter measurements taken prior to the activity outside the standard protocol were included in the statistical analysis. This approach aimed to assess the actual pupil diameter achieved solely with Mydrane.

The enrolment was carried out among consecutive patients hospitalized in the clinical hospital for cataract surgery. Overall, 296 patients came to the initial examination (V1), 105 of whom were qualified for cataract surgery with Mydrane (V2). Finally, data from 103 patients (103 eyes) were analyzed in the study, as 2 patients did not attend V2 or were due to missing data. These were divided into 3 groups: cataract and DM (C+DM; *n* = 35), cataract and PXF (C+PXF; *n* = 32), and cataract with no DM or PXF (C, reference group; *n* = 36).

### 2.2. Study Design

Pupil diameter measurements obtained during 2 subsequent visits were used. During the first visit (V1), from 1 to several days before the surgery, 2 measurements with a NeurOptics pupilometer were made, of which the second, V1-2 (20–30 min after the third administration of ME at intervals of about 10 min) was analyzed in the presented study. During this visit, information about coexisting diseases, medical treatment, and ophthalmic procedures was also collected (Table 1). In the second visit (V2), on the day of the cataract surgery, 5 measurements were taken, 2 of which were analyzed in the study: V2-2 (30–40 s after Mydrane administration to the anterior chamber) and V2-3 (just before capsulorhexis, after viscoelastic administration). Due to technical capabilities and the necessity to maintain sterile conditions, the measurement of pupil diameter during the surgical procedure was conducted using a sterile medical ruler—either paper or stainless steel—with an accuracy of 0.5 mm. The designated measurement time took into account the necessary duration to achieve a pupil diameter that does not respond to changes in light intensity.

The effectiveness of Mydrane during phacoemulsification procedures performed in everyday medical practice was assessed in patients with cataract coexisting with DM (without PXF) and with cataract coexisting with PXF (without DM). On the basis of the available literature [15,28,31], effective mydriasis was defined as obtaining a pupil diameter no smaller than 6.0 mm in V2-3 without the use of any additional devices dilating the pupil. The stage of capsulorhexis during phacoemulsification was considered to be particularly important because the quality of its performance determines the further course of the operation and is closely related to the quality of mydriasis. Additionally, it was analyzed whether the effect of the pharmacological mydriasis after Mydrane injection during phacoemulsification was comparable with that in the reference group in those patients.

Furthermore, the pupil diameter obtained after the use of standard mydriatics and after the Mydrane injections were compared in the same patients. For objective comparison, the reference point was V1-2, determined 30–40 min after the instillation of ME, and V2-2, taken 30–40 s after Mydrane administration.

In the center where the studies were conducted, each patient was routinely locally anesthetized with 2% lidocaine gel before cataract surgery. Additionally, analgosedation was administered—patients received 0.5–1.0 mg of midazolam and 0.05–0.1 mg of fentanyl.

### 2.3. Statistical Methods

Two-tailed *p* < 0.05 was assumed to be statistically significant. The calculations were performed with the Statistica v.13 program.

Continuous parameters are presented as average (±standard deviation) or median and interquartile range (Q1–Q3), depending on the data distribution. Ranges of values of continuous variables (minimum-maximum) are also allocated. The selected graphs show 95% confidence intervals for the averages. The qualitative parameters are depicted in the form of the number of observations and percentages. The visual evaluation of the distribution and the Shapiro–Wilk test were used to assess the normality of the distributions of continuous variables. The significance of differences between the averages was tested using the Student’s *t*-test or Mann–Whitney U test, depending on the data distribution. In order to compare the continuous parameters between more than 2 groups, the ANOVA or the Kruskal–Wallis H test, depending on the data distribution, was used. ANOVA in repeated measures designs served to compare the measurements at the subsequent visits. Tukey’s honest significance test was applied in post-hoc analysis for continuous variables. Qualitative parameters were tested with the χ^2^ (chi-square) test with an optional Yates’s correction, which was utilized if any of the expected frequencies were <5. In the case of multiple comparisons for qualitative features, the classic Bonferroni correction was applied, which consisted of multiplying the test probability value by the number of multiple comparisons performed.

## 3. Results

### 3.1. The Effectiveness of Mydriasis

In V2-3, in each of the groups C, C+DM, and C+PXF (*n* = 36; *n* = 35; *n* = 32, respectively), all patients had a pupil diameter ≥ 6.0 mm. Thus, the evaluation of the efficacy of pharmacological mydriasis after the intracameral administration of Mydrane revealed that in 100% of the examined patients (*n* = 103), mydriasis was effective (Table 2).

### 3.2. Pupil Diameter Measurements

**Figure 1:**
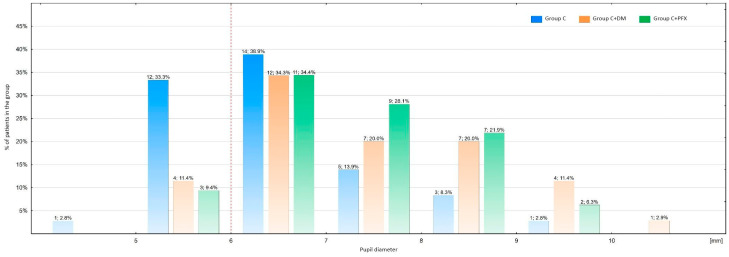
Mydriasis achieved in V2-2 in the examined groups. C: cataract; C+DM: cataract and diabetes mellitus; C+PXF: cataract and pseudoexfoliation syndrome. Above the bars, the number of patients and percentage of patients in the group are indicated. C—with cataract; C+DM—with cataract and diabetes; C+PXF—with cataract and pseudoexfoliation syndrome.

In V2-2 (Figure 1), the average pupil diameter for all examined patients was 6.8 mm. Comparative analysis of mean pupil diameter measurements in V2-2 (Figure 1) in individual groups (C vs. C+DM vs. C+PXF) revealed that the average pupil diameter in the C+DM group (7.1 mm; *n* = 35; *p* = 0.004) and the C+PXF group (7.0 mm; *n* = 32; *p* = 0.008) was significantly larger compared to the C group (6.2 mm; *n* = 36) (Table 2).

**Figure 2:**
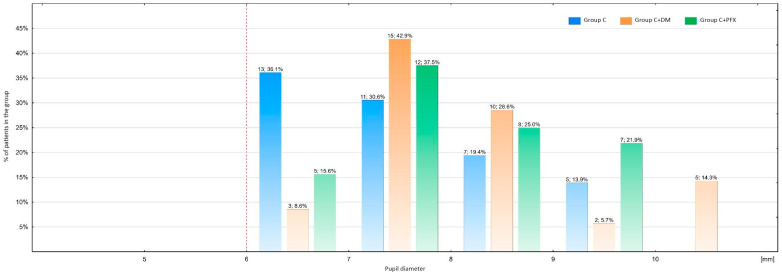
Mydriasis achieved in V2-3 in the examined groups. C: cataract; C+DM: cataract and diabetes mellitus; C+PXF: cataract and pseudoexfoliation syndrome. Above the bars, the number of patients and percentage of patients in the group are indicated. C—with cataract; C+DM—with cataract and diabetes; C+PXF—with cataract and pseudoexfoliation syndrome.

In V2-3 (Figure 2), the average pupil diameter in all examined patients was 7.7 mm. In V2-3, a statistically significant difference was observed (*p* = 0.04) between group C (*n* = 36), where the smallest pupil dilation was obtained (7.4 mm), and group C+DM (*n* = 35), where the largest pupil dilation was noted (8.0 mm). In the C+PXF group (*n* = 32), the measurement (7.8 mm) did not differ significantly (*p* = 0.30, *p* = 0.64, respectively) from that in the other groups (Table 2).

**Figure 3:**
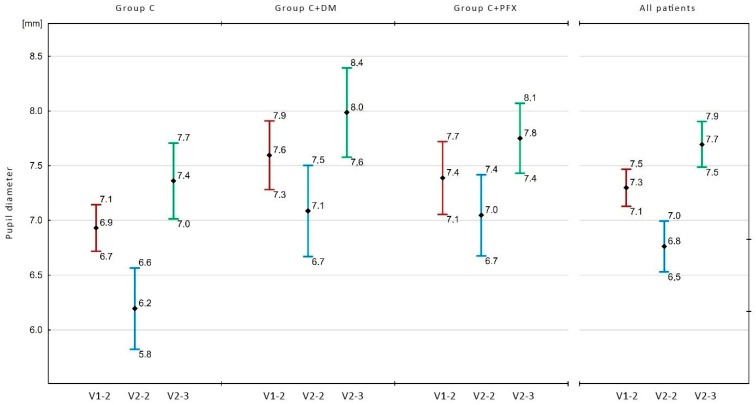
Pupil diameter during particular visits in the examined groups. C: cataract; C+DM: cataract and diabetes mellitus; C+PXF: cataract and pseudoexfoliation syndrome. Whiskers show 95% confidence intervals for the averages shown. V1-2—measurement 30–40 min after administration of standard mydriatics; V2-2—measurement 30–40 s after Mydrane administration; V2-3—measurement before capsulorhexis after administration of viscoelastic; C—with cataract, C+DM—with cataract and diabetes; C+PXF—with cataract and pseudoexfoliation syndrome.

The average pupil diameter in all examined patients (*n* = 103) was significantly larger (*p* ≤ 0.001) after the administration of ME during V1 in V1-2 (7.3 mm) than after the application of Mydrane during V2 in V2-2 (6.8 mm). The comparison of the pupil diameter during particular visits in the investigated groups (C, C+DM, C+PXF) revealed that the pupil diameter in V1-2 was significantly larger than in V2-2 in the C group (6.9 mm vs. 6.2 mm; *p* < 0.001) and in the C+DM group (7.6 mm vs. 7.1 mm; *p* = 0.001), but not in the C+PXF group (7.4 mm vs. 7.0 mm; *p* = 0.08) (Figure 3).

### 3.3. Adverse Events

During V1, ME caused discomfort in some patients, which they described as ‘pinching’ and ‘burning’ in the eye. There were no other adverse events.

No ophthalmic or general adverse events resulting from the use of Mydrane were observed during V2. Intraoperative complications included moderate intraoperative floppy iris syndrome (*n* = 1) [32], anterior chamber shallowing (*n* = 1), lack of patient cooperation with the surgeon (*n* = 2), and unstable mydriasis (*n* = 1). In the case of unstable mydriasis, after V2-3, the surgeon decided to administer epinephrine to maintain pupil dilation in a patient without PXF and DM.

## 4. Discussion

The safe course of cataract surgery, and thus the reduction of intra and postoperative complications, depends mainly on the effectiveness and stability of the obtained pharmacological mydriasis [28]. Mydriasis is considered successful when the anticipated pupil dilation is achieved through the chosen method. Most authors [15,28,29] consider a pupil diameter of ≥6.0 mm as the optimal mydriasis for cataract phacoemulsification. It allows proceeding with safe phacoemulsification for the patient and creates convenient conditions for the surgeon.

Mydrane is the first standardized combination of mydriatics and anesthetic to be administered into the anterior chamber during cataract phacoemulsification to obtain mydriasis. Phase II and III studies [28,29] have shown that intracameral Mydrane injection is safe and results in rapid, effective, and stable mydriasis in patients with uncomplicated cataract. Such diseases as DM or PXF may constitute factors of increased risk in obtaining effective mydriasis during cataract phacoemulsification. Although many studies have shown that diabetic patients have small pupils and are less responsive to mydriatics [33,34], according to Coblentz et al. [35], diabetics can achieve similar satisfactory pupil dilation as non-diabetic patients if an appropriate combination of drugs is provided. Accordingly, in this study, the authors assumed that Mydrane was likely to be as effective in patients with diseases affecting iris function as in those without them, owing to the direct administration of active substances, presenting superadditive properties in the immediate vicinity of iris receptors.

In this study, the effectiveness of mydriasis was defined as achieving a pupil diameter of ≥6.0 mm just before capsulorhexis, following the administration of Mydrane and viscoelastic agents, without the use of any additional dilating agents. Each patient obtained a pupil diameter of ≥6.0 mm in V2-3 after Mydrane application, which ensured 100% effectiveness of mydriasis in the entire group in the initial stages of phacoemulsification (Figure 2). All cataract patients (*n* = 103), both with and without DM or PXF, obtained an average pupil diameter of 7.7 mm in V2-3. It should be emphasized that in phase III, in contrast to the presented research, patients with diseases such as PXF and DM treated with insulin were excluded. Therefore, only the results from the C group, without DM or PXF, are comparable. In a phase III study, after Mydrane administration [29], in 96.8% of patients, the average pupil diameter was ≥6.0 mm just before capsulorhexis (after the administration of a viscoelastic), while in patients from the C group in this study, the effectiveness of mydriasis was also very high and amounted to 100%.

The present paper widens the knowledge concerning effective mydriasis achieved with Mydrane by referring to 2 separate groups of patients with particular diseases: cataract and DM (without PXF) and cataract and PXF (without DM). Patients with these diseases constitute a vast majority of cataract patients.

In a study by Labetoulle et al. [36], attention was drawn to the importance of effective mydriasis in patients with cataract and DM; however, in contrast to the C+DM group of the present study, only patients with cataract and non-insulin-dependent DM were included in the study conducted by Labetoulle et al. [36]. Despite this, the effectiveness of mydriasis was very high in both studies. In this study, it reached 100% (*n* = 35/35), while in that by Labetoulle et al., it equaled 91.7% (*n* = 22/24).

Paradoxically, in the C+DM group, the pupil diameter in V2-3 was significantly larger than in the reference group C (8.0 mm vs. 7.4 mm). The possible reasons for such a reaction to mydriatics in patients with DM may vary [37,38]. On the one hand, it can be expected that local iris damage resulting from ongoing DM will reduce the response to any mydriatic drug [39]. On the other hand, on the basis of the information obtained during studies on the small pupil in Horner’s syndrome, one would expect that iris denervation improves the response to phenylephrine (a directly acting sympathomimetic amine), which dilates the pupil by activating α-adrenergic receptors in the pupil retractor muscle [40,41].

Similar observations were made in a study by Bremner and Smith [42], in which the pupil dilated paradoxically more in patients with DM (treated with insulin) than in those without DM. Although the mechanism of obtaining a significantly larger pupil diameter in the C+DM group only is not fully understood, such a reaction was most likely associated with an excessive response to phenylephrine eyedrops owing to the denervation of the iris sympathetic fibers in diabetic patients [37,43]. It appears that the response to mydriatics varies depending on the etiology of the small pupil [44]. Compared with this study, Nuzzi et al. [15] obtained lower mydriasis efficacy (69.5% of patients; *n* = 32) after administering Mydrane in a group of patients with cataract and systemic diseases. In turn, Schulz et al. [45] and Nazim-Lipski et al. [46] observed that in cataract patients with coexisting diseases, these diseases did not significantly affect pupil diameter after Mydrane administration. As patients with various diseases were included in these studies, it is difficult to draw unambiguous conclusions about a particular disease that affects the study result (including the effect of α1-blockers).

Although limited, the literature includes individual reports examining alterations in pupil diameter and the effects of pharmacological mydriasis in eyes affected by PXF syndrome. This investigation was conducted by Yiğit et al. [47] and Tekin et al. [48], both of whom confirmed the presence of structural changes in the iris associated with PXF syndrome, which influenced pupil diameter and, consequently, the achieved mydriasis. When analyzing the available literature, no information was found on the efficacy of mydriasis after Mydrane administration in a selected group of patients with PXF, especially since PXF was among the exclusion criteria in phase II and III studies [28,29]. Nuzzi et al. [15] found a lower efficacy of mydriasis in 76.2% of patients in the group, which also included people with PXF. However, they did not provide information on the percentage of PXF patients or the percentage of other diseases affecting the achievement of effective mydriasis. In the present study, the average pupil diameter in V2-3 was 7.8 mm in the C+PXF group and did not significantly differ from the average pupil diameter of 7.4 mm in the C group. In turn, in the study by Nuzzi et al. [15], the average pupil diameter in the PXF group was significantly smaller just before capsulorhexis than in patients with uncomplicated cataract. The results suggest that the use of Mydrane provides effective mydriasis in patients with cataract and PXF, which may significantly affect the safety and effectiveness of phacoemulsification treatment. 

Ripa et al. [11], like the authors of this study, observed that due to the rigorous inclusion and exclusion criteria of the phase III study, the results could not be generalized to the entire population of patients undergoing cataract surgery. Therefore, in their study, they verified the effectiveness of mydriasis in a selected group of patients with primary open-angle glaucoma, which has a worldwide overall prevalence of 2.4%. As mentioned earlier, glaucoma can also impact the occurrence of difficulties in achieving adequate mydriasis during cataract surgery. They found that the mean pupil size before capsulorhexis (after viscoelastic administration) was 6.45 mm in patients after ME administration, 6.46 mm in patients with Mydriasert application, and 6.16 mm when Mydrane was used (*p* = 0.86). The main pupil size using all three different mydriatic drugs was higher than the critical value of the required 6.0 mm, so the effectiveness of mydriasis in patients with primary open-angle glaucoma was also considered effective.

Both DM and PXF present challenges in achieving and sustaining effective mydriasis. Thus, as proposed by Mori et al. [43], it becomes imperative to adopt the most efficient method of pupil dilation tailored to the specific underlying cause. The advantage of this study lies in its establishment of a well-defined cohort (a homogeneous group of patients) comprising individuals with DM but without PXF and those with PXF but without DM, all of whom did not use α1-blockers. This approach allows for a real assessment of mydriasis effectiveness after Mydrane administration in patients with these particular diseases.

From a practical and clinical perspective, the effectiveness of mydriasis after administering Mydrane did not show significant differences in the C+PXF and C+DM groups compared to the C group. Thus, with the probability adopted in the methodology of this study, it can be concluded that in the population of cataract and diabetic patients, as well as in the population with cataract and PXF, the use of Mydrane will provide effective mydriasis. The data gathered in this study on intracameral administration of Mydrane are promising, especially in relation to patients with the studied diseases.

Additionally, because a pupil diameter of ≥6.0 mm was used as the inclusion criterion (not ≥7.0 mm, as in a phase III study [29]), the potential of Mydrane has been demonstrated with good results in an even wider group of patients (with a pupil diameter of 6.0–7.0 mm obtained during the V1-2 measurement), as in a study by Kęcik et al. [49].

In the available literature, various studies compare pupil diameter measurements following the application of ME and intracameral mydriatics at different phases of phacoemulsification surgery (especially before or after viscoelastic administration). Additionally, these studies describe moderately different intracameral substances. Nevertheless, there is a trend for the pupil diameter to become larger after the administration of ME than after using intracameral ones [28,29,36,50,51] as well as after using Mydriasert [11,52]. 

In the study by Morgado et al. [52], a comparison was conducted between extracameral mydriatics, such as ME and Mydriasert, and intracameral mydriatics, including a mydriatic cocktail comprising 1% lidocaine, 0.1% cyclopentolate, and 1.5% phenylephrine. The measurement of pupil size before the administration of viscoelastic was 8.1 mm, 8.2 mm, and 6.3 mm, respectively. Due to this and the diverse medical conditions of the patients in that study, it is understandable that the comparison should encompass all patients during the V1-2measurement (7.3 mm) for extracameral administration and V2-2 (6.8 mm) for intracameral mydriatics comparison.

Additionally, in the study by Faria et al. [26], the pupil size after the use of extracameral mydriatic—Mydriasert and intracameral mydriatic—Mydrane, before the administration of viscoelastic was compared (respectively, 7.21 mm; 6.35 mm). Patients with DM, PXF, and other diseases were excluded. In this case, the presented measurements should be compared with the measurements from group C: V1-2 (6.9 mm) for extracameral administration and V2-2 (6.2 mm) for Mydrane administration comparison.

In all three studies, regardless of the route of administration of mydriatics, mydriasis was effective, measuring >6.0 mm. Although mydriasis after the administration of ME and Mydriasert was greater than after intracameral administration, the authors disagree with Morgado et al. [52] that the latter method should be considered only in selected cases. Authors do agree, however, with Faria et al. [26] that it remains comfortable for the performance of capsulorhexis, as it helps save preoperative time and provides additional anesthesia for cataract surgery. The administration of mydriatics to the anterior chamber supports a lower drug dose, translating into a lower risk of adverse effects, reduced involvement of medical personnel (an economic aspect), and greater patient comfort [22]. However, these particular concerns are not the primary focus of this study.

Also, in this study, the pupil diameter after ME application was statistically significantly larger than after the intracameral administration of Mydrane (*p* < 0.001). The pupil diameter in the V1-2 measurement for the entire study group averaged 7.3 mm, whereas it registered 6.8 mm in the V2-2 measurement. The authors noted similar differences when analyzing each individual group. Notably, in groups C and C+DM, the average pupil diameter in the V1-2measurement (6.9 mm and 7.6 mm, respectively) was statistically significantly larger (*p* < 0.001; *p* = 0.001, respectively) than in the V2-2 measurement (6.2 mm and 7.1 mm, respectively). In the C+PXF group, the difference in pupil diameter between the V1-2 measurement and the V2-2 measurement, though not statistically significant (*p* = 0.08), approached the threshold of statistical significance. Furthermore, in the V1-2 measurement, the pupil diameter was larger than in the V2-2 measurement (7.4 mm vs. 7.0 mm). Consequently, mydriasis after ME instillation was larger but ensued slower than mydriasis after Mydrane administration, which ensued faster.

However, the limitation of this study was the measurements of pupil diameter using different measurement tools during V1 and V2. The measurements performed after the administration of both ME and SCIMA provided, as the authors believe, a reliable comparison of the effectiveness of mydriasis, as the patients were the same in both measurements and had the same diseases that could affect pupil dilation during phacoemulsification. 

## 5. Conclusions

The authors of this study maintain that Mydrane proves to be an effective agent for dilating the pupil, especially in patients with coexisting diseases that may impact the achievement of the required pupil diameter, such as PXF and DM. What is more, Mydrane seems to be suitable for patients with DM in which the pupil diameter might be paradoxically significantly larger due to the denervation of the iris sympathetic fibers in diabetic patients [37,43]. Current research strongly supports the notion that intracameral mydriatics provide a favorable balance between benefits and risks. One of them is minimizing the drug dosage directly around the iris receptors, which reduces the occurrence of side effects—both on the ocular surface and systemically. Additionally, effective mydriasis after applying Mydrane can be achieved immediately, without the need to wait, as in the case of ME or Mydriasert. When carefully assessing the actual advantages and disadvantages, they emerge as a genuine alternative to mydriatics in eyedrop [21] or Mydriasert [26]. 

## Figures and Tables

**Table 1:** Clinical characteristics of all examined patients (*n* = 103) included in the study.

Characteristics of the Whole Group (*n* = 103)		Values
Age [years]		71.7 (±7.45)
	Min. Max.	53.0 87.0
Sex	Female	85 (82.5%)
	Male	18 (17.5%)
Operated eye	Right	50 (48.5%)
	Left	53 (51.5%)
CDVA [decimal (logMAR)]	≥0.5 (+0.3)	46 (44.7%)
	<0.5 (+0.3)	20 (19.4%)
	<0.3 (+0.5)	24 (23.3%)
	<0.1 (+1.0)	2 (1.9%)
	<0.05 (+1.3)	11 (10.7%)
Glaucoma		25 (24.3%)
Dry AMD		3 (2.9%)
Hypertension		82 (79.6%)
PXF		32 (31.1%)
DM		35 (34.0%)
Patients with DM	Oral medications only	20 (57.1%) *
	Insulin therapy	15 (42.9%) *
	Diabetic retinopathy	6 (17.1%) *

Values are presented as average (±standard deviation) or number of observations (percentage). * Percentage of diabetic patients. AMD: age-related macular degeneration; CDVA: corrected distance visual acuity; DM: diabetes mellitus; max.: maximum; min.: minimum; PXF: pseudoexfoliation syndrome.

**Table 2:** Comparison of the average pupil diameter between the examined groups.

Second Visit		Pupil Diameter [mm]			*p*	Post-Hoc Tests		
		(A) Group C (*n* = 36)	(B) Group C+DM (*n* = 35)	(C) Group C+PXF (*n* = 32)		*p*: A vs. B	*p*: A vs. C	*p*: B vs. C
V1-2	20–30 min after standard mydriatics	6.9 (±0.63)	7.6 (±0.91)	7.4 (±0.92)	0.002 *	0.003 *	0.08	0.58
V2-2	30–40 s after Mydrane	6.2 (±1.1)	7.1 (±1.22)	7.0 (±1.03)	0.001 *	0.004 *	0.008 *	0.99
V2-3	Before capsulorhexis	7.4 (±1.03)	8.0 (±1.18)	7.8 (±0.89)	0.04 *	0.04 *	0.30	0.64

Values are presented as average (±standard deviation). * Statistical significance. C: cataract; C+DM: cataract and diabetes mellitus; C+PXF: cataract and pseudoexfoliation syndrome.

## Data Availability

Data are available from the corresponding author upon reasonable request.
